# Associations of working conditions and chronic low-grade inflammation among employees: a systematic review and meta-analysis

**DOI:** 10.5271/sjweh.3982

**Published:** 2021-10-31

**Authors:** Helena C Kaltenegger, Linda Becker, Nicolas Rohleder, Dennis Nowak, Matthias Weigl

**Affiliations:** 1 Institute and Clinic for Occupational, Social and Environmental Medicine, University Hospital, LMU Munich, Munich, Germany; 2 Chair of Health Psychology, Institute of Psychology, Friedrich-Alexander University Erlangen-Nürnberg, Erlangen, Germany; 3 Institute for Patient Safety, University Hospital Bonn, Bonn, Germany

**Keywords:** health, immune system, inflammatory biomarker, information and communication technology, job, occupational stress, work

## Abstract

**Objectives:**

Chronic low-grade inflammation has been identified as a key pathway linking stress experience to human health. However, systematic evaluations on the relationship of work stress and immune function are scarce and predominantly based on cross-sectional studies. We performed a systematic review and meta-analysis of prospective studies on associations of working conditions and inflammatory biomarkers.

**Methods:**

In line with our previously established study protocol and the PRISMA-guidelines, we systematically searched electronic databases for prospective studies on working conditions as well as workplace interventions and inflammatory markers in employees. We classified studies (by design, type of exposure/intervention, outcome) and performed rigorous risk-of-bias assessments. Studies were summarized qualitatively, and a meta-analysis was conducted.

**Results:**

We identified 23 eligible studies (N=16 432) with a broad scope of working conditions and inflammatory markers. For interventional designs, we differentiated between individual-directed/behavioral (including physical and mental) and organization-directed/structural interventions. Workplace physical exercise interventions were associated with a decrease in C-reactive protein (k=5; d=-0.61; P<0.001). For other workplace interventions, ie, mental and organizational/structural, results were inconclusive. Concerning observational studies, dimensions of the job demand–control(–support) model were most frequently investigated, and results showed weak – if any – associations with inflammatory markers.

**Conclusions:**

The research base was heterogeneous and high-level evidence was limited. More prospective studies are needed with broader consideration of work stressors and inflammatory markers. For practical occupational health management, exercise interventions are effective measures to reduce chronic low-grade inflammation.

Given the profound transformation of work in the age of digitalization, investigations into ramifications for employee health are of crucial importance. There is substantial evidence for associations between exposure to workplace-related stressors and risk of physical as well as mental morbidity, including cardiovascular diseases (CVD), metabolic conditions, depression, etc, and mortality (1–9). Over the past years, research on work stress has expanded the focus on job task characteristics [such as described in Karasek’s job strain model (10)] to organizational factors (such as working hours or organizational justice) and also broader labor market conditions (such as job insecurity) and their effects on employee health (11–14). Work stress is typically classified as chronic stress, ie, prolonged or repeated stress exposure, although there is no clear time point to differentiate between chronic and acute stressors (15–17).

In general, the human stress response involves –besides the engagement of the main stress systems [ie, autonomic nervous system (ANS) and hypothalamic-pituitary-adrenocortical (HPA) axis] – complex effects on the immune system most importantly with up-­regulation of inflammatory pathways and down-regulation of cellular immunity (18–21). While in the short term these adaptations serve protective functions (22, 23), sustained and systemic low-grade inflammation implies a dysregulation of the immune system and has been suggested as a mediator in the pathogenesis of chronic diseases (20, 24–27). Particularly, inflammatory biomarkers, such as C-reactive protein (CRP), Interleukin-6 (IL-6), but also leukocytes, are involved in the atherosclerotic process (28, 29).

Reviews and meta-analyses in the field of psychoneuroimmunology demonstrate a large body of evidence that psychosocial distress affects immunological and inflammatory activity (17, 30–32). Specifically for work stress, studies reported associations between adverse working conditions and chronic low-grade inflammation in employees, as for instance effort–reward imbalance (ERI) (33), long working hours (34), job strain and poor social support (35, 36). However, two pivotal limitations arise from the current literature base.

First, conclusive and systematic syntheses of the current knowledge base as well as quantitative aggregation of effects of work-related stress on employees’ chronic low-grade inflammation are scarce. Previous reviews and meta-analyses have focused on associations of psychosocial job stress (37, 38) and herein particularly ERI (39) with immune and inflammatory markers. Those reviews have the limitation of including a significant number of cross-sectional studies, what limits inferences concerning cause-effect relationships.

Secondly, collated evidence is lacking with regard to effects of other work exposures – besides the commonly studied psychosocial work factors – on employees’ immune function. With the ubiquitous and ever-increasing use of information and communication technologies (ICT) in the workplace, associated risks of professionals’ stress experience have become a phenomenon of growing scholarly interest. Human interaction with ICT at work is suggested as a potential source of negative psychological and biological sequelae for health and well-being (40, 41). Yet, as far as we are aware, knowledge gaps exist with respect to how working conditions related to the omnipresence and use of ICT and concomitant new demands, but also resources for employees (42) have effects on physiological stress responses in terms of low-grade inflammation.

A review based on prospective studies allows for conclusions on a higher level of evidence and for inferences concerning temporal order and direction of effects in the interplay of workplace stressors and inflammatory reactivity as a risk factor to serious long-term diseases (43). Beyond temporal sequence, ie, the exposure precedes the outcome, one important indicator of causation is reversibility, ie, mitigation of work stress reduces the health risk (13, 44). The consideration of interventional studies with high-quality designs [ie, randomized trials (45)] in addition to observational prospective studies, may therefore not only provide a more complete summary of the evidence, but also deeper insights into potential cause-effect relationships between work stressors and inflammatory markers.

We conducted a systematic review and meta-analysis to determine the present evidence base on prospective associations between various working conditions and chronic low-grade inflammation in employees. More specifically, we aimed to (i) systematically summarize the current research base and establish quantitative estimations of associations. Furthermore, we sought to (ii) detect studies on ICT use at work and inflammatory markers. Lastly, we aimed to (iii) identify and evaluate workplace-related interventions to decrease inflammation.

## Methods

### Protocol and registration

First, a systematic review protocol was developed and published (46). The review was registered in the PROSPERO-database (registration ID: CRD42020166887). It adheres to the Preferred Reporting Items for Systematic Reviews and Meta-analyses (PRISMA) guidelines (47; PRISMA-checklist upon request). No major deviances from the original protocol were undertaken. Minor adaptations related to the use of software, waiver of graphical synthesis, and use of AMSTAR-2 instead of GRADE (for details, please see below).

### Eligibility criteria

We searched for studies on associations between working conditions and inflammation fulfilling the following PECOS/PICOS-criteria: *Participants (P)*: adult employees/workers/professionals. Clinical samples with particular diagnoses as well as specific professional groups, like military personnel, athletes, artists, and students were excluded. *Exposures/interventions (E/I):* all kinds of working conditions and workplace-related interventions, including psychosocial, mental, and physical. There were no a-priori restrictions by type of workplace intervention, meaning all measures aiming at occupational health promotion or well-being on the job, conducted on or off site as well as during or outside working hours, were considered. Studies on environmental hazards, ie, chemical or biological agents and extreme heat, as well as nutritional, pharmaceutical, or nutraceutical interventions were not eligible. Furthermore, we excluded studies on shiftwork and exclusive shiftwork samples (48, for a review) as well as studies on socioeconomic status as exposure. A particular objective of our review were effects of work-related use of digital technologies and media, defined as all electronic devices (hardware), applications (software), and means of communication, such as computers, mobile phones, messaging systems, autonomous systems, etc. *Comparators (C):* workers not or exposed to a lower extent to working conditions/workplace interventions of interest. *Outcomes:* pre-defined biomarkers of inflammation within three main categories (cells, plasma molecules, intracellular processes) measured in blood or saliva (see supplementary material, www.sjweh.fi/article/3982, table S1). *Study design (S)*: prospective studies with at least one follow-up measure, ie, at least two consecutive measurements of the inflammation outcome. We included observational (eg, cohort) and interventional studies, ie, randomized controlled trials (RCT) and non-randomized studies of interventions (NRSI, eg, before-after studies). Laboratory or simulation studies were not eligible. We included original research articles in the languages English or German published in peer-reviewed journals from 1982 until present. Conference proceedings, study protocols, and theses were excluded.

### Information sources

As primary information source, we conducted a systematic search in five electronic databases, including PubMed/MEDLINE, Embase, PsycINFO, Web of Science, and Cochrane’s CENTRAL. Our search was finalized in November 2020. In addition, we performed citation searching of included studies in Google Scholar (forward search) and hand-searching of reference lists of included studies and relevant reviews (backward search).

### Search and study selection

We developed a four-tier search string comprising a broad spectrum of terms related to the specified PECOS/PICOS elements (see also 46). The four blocks were linked with the Boolean operator “AND” and within the blocks the terms were combined by “OR”. The screening procedure of retrieved records was conducted in Rayyan (49). Two reviewers independently performed systematic and stepwise assessment of eligibility (HK, MW). First, titles and abstracts were screened and then full-texts were assessed. The title and abstract screening were pre-tested, in order to ensure a joint understanding of the eligibility criteria. Discrepancies and uncertainties were resolved by discussion as well as consultation of other review members until consensus was reached.

### Data collection process and data items

Two reviewers (HK, MW) extracted data of included studies in a pilot-tested Excel sheet (table S2) that was based on the Cochrane Consumers and Communication Group’s template (50). In case of missing information, we contacted authors. We obtained additional data from four authors. For multiple publications of identical data, only one study with longer follow-up period was included. Information was extracted on (46): study characteristics (authors, year, design, location, follow-up, occupational setting); *P*=participants’ professional characteristics, age, gender, ethnicity, health-related variables, sample size, recruitment method, relevant inclusion/exclusion criteria; *E/I and C*=type and description of working condition/workplace intervention and comparators, theoretical foundation, and assessment; *O*=type and assessment of outcomes; statistical analyses, results, and moderators/control of confounders. Where reported, we extracted data from adjusted models for baseline biomarker levels and/or important covariates such as age or sex. After data extraction, professional samples were grouped into occupational settings based on the ILO classification of industries and sectors (51).

### Risk of bias in individual studies

Two reviewers (HK, MW) performed standardized risk of bias (RoB) assessments, and systematic evaluations were established after consensus. For RCT, the updated version of the commonly used Cochrane risk-of-bias tool (RoB 2; 52), and for NRSI, ROBINS-I was applied (53). Observational studies were assessed with the Quality of Reporting of Observational Longitudinal Research checklist (54). A summary score was calculated with higher scores indicating better quality (54).

### Synthesis of results

Synthesis of results comprised three steps: First, we clustered studies by design, exposure/intervention, and outcome. Concerning exposures/interventions, we applied our pre-defined classification system: studies were categorized based on underlying theoretical models and specific exposure features: mental versus non-mental, acute versus chronic, investigation of digital technology use (for definitions, see above and 46). Second, we provided a qualitative summary of all included studies in narrative and tabular format. In addition, main results were visualized by means of arrows indicating direction of effects. Third, where possible, we performed quantitative syntheses of sufficiently similar studies. Otherwise, results were summarized narratively for at least two studies within one cluster. A random-effects meta-analysis was conducted utilizing Meta-Essentials (55). Heterogeneity was evaluated using Q statistic with p-value and I^2^ statistic. In case of low heterogeneity, additionally a fixed-effects model was applied. As the majority of studies were based on controlled designs with repeated measurements, we chose an effect size that accounts for pre-post changes in different groups. In particular, we calculated the recommended pretest-posttest-control group effect size *d*_ppc2_, according to the following formula (56, 57):


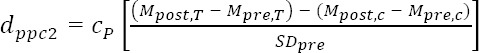



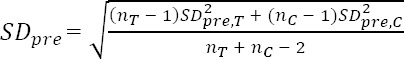



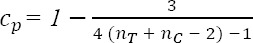


(*T*=treatment group; *C*=control group)

For the interpretation, the operational definition by Cohen (58) of d-values of 0.2, 0.5, and 0.8 representing small, medium, and large effect sizes was used. As a sensitivity analysis, we excluded studies attributed a high RoB.

### Risk of bias across studies

In order to assess RoB across studies, we used funnel plots, tests for funnel plot asymmetry (59), and the Trim-and-Fill procedure (60, 61). Furthermore, we applied the appraisal instrument AMSTAR-2 for evaluation of the quality of our review (62).

## Results

### Study selection

**Figure 1:**
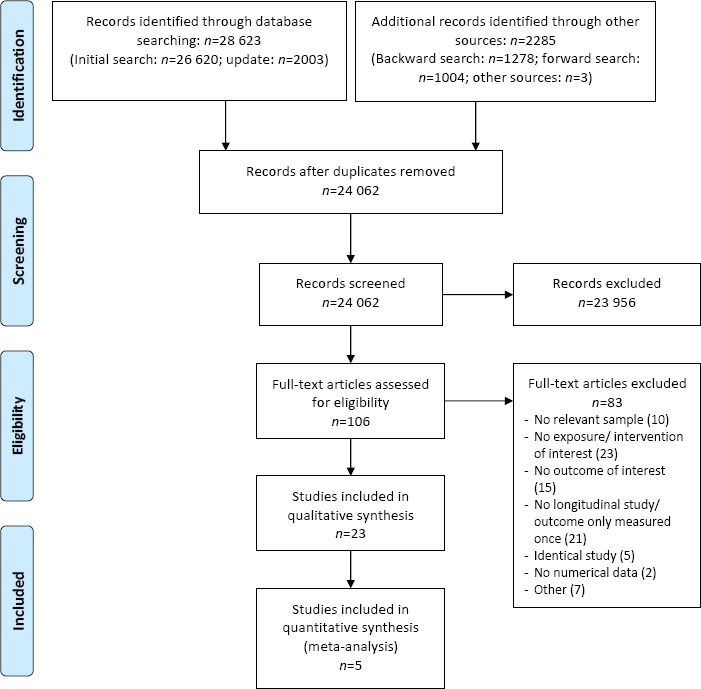
PRISMA flow chart according to Moher et al (47).

The database search yielded a total of N=28 623 records. After removal of duplicates, 24 062 records remained and were screened by title and abstract; 23 956 records were discarded. Besides, we identified 2285 additional records and 3 reviews relevant to our research question, which were screened for further eligible studies (38, 63, 64). In total, 106 full-texts were assessed in detail, of which 83 studies did not meet our inclusion criteria (list of excluded studies upon request). Eventually, 23 studies were included in the qualitative and 5 studies in an additional quantitative analysis. A PRISMA flow diagram depicts the study selection process (figure 1).

### Study characteristics

The characteristics of all included studies are presented in table 1. There were two major clusters of study designs: 16 interventional studies, including 8 RCT (65–72) and 8 NRSI (73–80) as well as 7 observational studies (81–87). The majority of studies (k=13) (65–68, 70, 73, 75–77, 79, 80, 84, 86) came from Europe, 4 from Asia (71, 72, 83, 87), and 2 from the USA (69, 78) (location in 4 studies not specified). In sum, data from N=16 432 (including dropouts, see table S2) participants were included in our review. Samples were based on different occupational settings, most frequently public service (k=7) (68, 70, 73, 78, 82, 84, 86), followed by health services (k=5) (65, 67, 69, 77, 85). The majority of studies excluded employees with particular diseases, such as CVD, diabetes, inflammatory conditions, and/or use of specific medication (table S2, for more details). We found high heterogeneity in studied work-related exposures/interventions. According to our pre-defined scheme for model- and feature-based classification of working conditions (46), we retrieved 5 studies that were based on established job stress models, including job demands and resources like job control, decision latitude, and workplace social support (73, 82, 83, 86, 87). Other or modified job stress models were examined in 2 studies (84, 85). Concerning specific features, we categorized 15 studies as investigating psychosocial or mental working conditions/interventions (65, 66, 69, 72–74, 76, 78, 80, 82–87) and 8 studies as assessing physical work-related exposures/interventions (67, 68, 70, 71, 75, 77, 79, 81). Furthermore, 3 studies (72, 74, 81) examined predominantly acute effects. Remarkably, we did not retrieve prospective studies on work-related ICT use, apart from one study reporting effects of a web-based workplace intervention (66). Concerning inflammatory outcomes, included studies covered all pre-defined categories (table S1). Most frequently surveyed were plasma molecules including CRP (67, 68, 70, 71, 73, 75, 77–80, 82–84, 86, 87) and cytokines (66, 67, 71, 73, 74, 79–82, 84–86). Inflammation-related processes on cell level, ie, leukocyte counts, were investigated in 3 studies (72, 85, 87). Intracellular processes, including gene expression (65, 72) and transcription factors (69), were also assessed in 3 studies (see tables 1 and S2).

**Table 1:** Study characteristics (N=23). [CRP=C-reactive protein; hs-CRP=high-sensitivity C-reactive protein; IFN-γ=interferon-gamma; IL=interleukin; JDC(S)=job demand–control(–support) model; MCP-1=monocyte chemoattractant protein-1 (MCP-1); NR=not reported; NRSI=non-randomized study of intervention; RCT=randomized controlled trial; TNF-α=tumor-necrosis-factor-alpha; W=women].

Study	Location	Design	Occupational setting	Sample size	Sex (% W)	Age, mean (SD)	Exposure/ Intervention	Outcome: category	Outcome: biomarker
Carlsson et al (73)	Denmark	NRSI	Public service	359	73.8	49.4 (0.4)	Workplace reorganization	Plasma molecules	CRP, fibrinogen, IL-6
Christian & Nussbaum (81)	NR	Observational	Mixed	24	20	32.4 (7.4); 26.4 (7.7) ^a^	Occupational physical demands	Plasma molecules	IL-6
Dich et al (82)	NR	Observational	Public service	7007 ^c^	30	49 (5.8)	JDC	Plasma molecules	CRP, IL-6
Dunne et al (65)	Ireland	RCT	Health services	42	NR	NR	Attention-based training program	Intracellular processes	Gene expression (TNF-α, IL-6)
Eguchi et al (83)	Japan	Observational	Mechanical and electrical engineering	2020	26.4	35.9 (10.4); 39.6 (10.1) ^b^	Workplace social support	Plasma molecules	hs-CRP
Elovainio et al (84)	England	Observational	Public service	4408	27.3	43.9	Organizational justice	Plasma molecules	hs-CRP, IL-6
Filaire et al (74)	NR	NRSI	Education	9	22.2	42.5 (2.4); 39.2 (2.5) ^b^	Lecturing to students	Plasma molecules	IL-10, IL-2, IL-4, TNF-α
Geus et al (75)	Belgium	NRSI	Financial services/ professional services	80	NR	49 (7); 43 (5) ^a^	Cycling to work	Plasma molecules	CRP
Hasson et al (66)	Sweden	RCT	Media; culture; graphical	303	38.3	NR	Web-based stress management system	Plasma molecules	TNF-α
Hewitt et al (67)	England	RCT	Health services	20	NR	42 (8); 41 (8) ^a^	Aerobic exercise program	Plasma molecules	CRP, TNFα, IL-6
Korshøj et al (68)	Denmark	RCT	Public service	116	75.9	45.3 (8.6)	Aerobic exercise intervention	Plasma molecules	Fibrinogen, hs-CRP
Lebares et al (69)	US	RCT	Health services	83 ^d^	48.2 ^d^	28.6 (2.7) / 28.7 (2.2); 27.4 (2.1) / 28.8 (2.4) ^a^	Enhanced stress resilience training	Intracellular processes	AP-1, NF-kappa B
Lee et al (85)	NR	Observational	Health services	41	100	29.9	Job stress	Cells, plasma molecules	White blood cells, IL-1β, IFN-γ, TNF-α
Magnusson Hanson et al (86)	England	Observational	Public service	4638	28	49.6 (6.0)	JDCS	Plasma molecules	hs-CRP, IL-6
Meyer et al (77)	Switzerland	NRSI	Health services	77	54.5	42.8 (9.0)	Promotional campaign of stair use	Plasma molecules	hs-CRP
Murphy et al (70)	Northern Ireland	RCT	Public service	37	64.9	41.5 (9.3)	Walking intervention	Plasma molecules	hs-CRP
Netterstrøm & Hansen (76)	Denmark	NRSI	Public transport	40	35	44.5; 43.5 ^a^	Outsourcing	Plasma molecules	Fibrinogen
Ramey et al (78)	US	NRSI	Public service	38	23.7	41.0 (7.6)	Resilience training	Plasma molecules	CRP
Shete et al (71)	India	RCT	Mixed	48	0	41.5 (5.2)	Yoga training	Plasma molecules	IL-6, TNF-α, hs-CRP
Shirom et al (87)	Israel	Observational	Mixed	1121	34.2	47 (~9)	JDCS	Plasma molecules, cells	hs-CRP, fibrinogen, white blood cell count
Skogstad et al (79)	Norway	NRSI	Construction	121	36	41.8 (12); 42.6 (12.5) ^b^	Leisure-time physical activity intervention	Plasma molecules	CRP, IL-6, TNF-α, MCP-1
Wachi et al (72)	Japan	RCT	Mixed	40	0	38.4 (8.4)	Recreational music-making	Intracellular processes; cells	IFN-γ mRNA, IL-2 mRNA, IL-6 mRNA, IL-10 mRNA, Leukocyte counts
Wultsch et al (80)	Austria	NRSI	Mixed	34	11.8	36.4 (8.9); 42.3 (11.2) ^a^	extended working periods	Plasma molecules	CRP, IL-6

a Age reported separately per group (control, intervention).

b Age reported separately for men and women.

c Only 39% of the initial sample (with complete biomarker data) were relevant to this review.

d Pooled data of two trials.

### Risk of bias within studies

The results of the RoB assessments (per domain and overall) for RCT and NRSI as well as of the quality of reporting assessment for observational studies are shown in table S3. All RCT were appraised to have “some concerns” regarding their overall RoB. Evaluations for NRSI are presented separately for controlled and uncontrolled studies and ranged from “serious” to “critical” overall RoB. For observational studies, on average 20 of the 33 checklist criteria were reported, leading to a mean summary score of 0.62 (range 0.41–0.70).

### Results of individual studies

In the following, results are described first for interventional and second for observational studies. For interventional designs, we further distinguished between individual/behavioral (ie, physical and mental) and organizational/structural interventions.

### Interventional studies

#### Individual/Behavioral Interventions

***Physical Interventions***. We found seven studies assessing effects of workplace physical activity/exercise interventions on inflammatory biomarkers, including five controlled (four RCT) and two uncontrolled studies. With regard to RCT, two studies examined effects of worksite aerobic exercise interventions in laboratory (67) and cleaning personnel (68). Murphy et al (70) investigated the influence of a walking program in civil servants. Respective control groups (CG) received either no training (67, 70) or lectures (68). In a further RCT, effects of a workplace-based yoga intervention were assessed in industry employees against a wait-list CG (71). A controlled study (ie, comparison to passive CG) investigated a cycling to work intervention in professionals of a health insurance company (75). Two uncontrolled NRSI were identified: a leisure time physical activity program in a road maintenance company initiated by the employer (79) and a promotional campaign for stair use in a hospital (77). All studies explored plasma molecules, most frequently CRP. Results per marker are presented in table 2.

**Table 2:** Workplace physical interventions and inflammatory biomarkers. Order of studies per biomarker, by risk of bias assessment, and alphabet. [CG=control group; CRP=C-reactive protein; IG=intervention group; IL-6=interleukin 6; TNF-α=tumor-necrosis-factor-alpha; ↓↓ Significant decrease in inflammatory biomarker following intervention (and no significant change/ increase in control); ↓ Tendency for decrease in inflammatory biomarker, non-significant (and no change/ increase in control); — No significant differences in inflammatory biomarker (between groups/ within group); ↑ Tendency for increase in inflammatory biomarker, non-significant (and no change/ decrease in control); ↑↑ Significant increase in inflammatory biomarker following intervention (and no change/ decrease in control)]

Marker and study	Type of physical intervention (duration, frequency)	Follow-up: period/number	Key findings	Direction of effect
**CRP**				
Hewitt et al (67) ^a^	Aerobic exercise (brisk walking/light jogging, 12 weeks, 4 times/week)	12 weeks/3	IG: significant reductions (week 1-4, 1-8), non-significant reduction (week 1-12) CG: no significant changes Between groups: no significant differences	↓↓ (week 1-4, 1-8) ↓ (week 1–12)
Korshøj et al (68) ^a^	Aerobic exercise (indoor biking/running, 12 months, 2 times/week)	12 months/1	IG: no significant changes CG: significant increase Between groups: significant difference	↓
Murphy et al (70) ^a^	Walking (8 weeks, 2 days/week)	8 weeks/1	IG: no significant changes CG: no significant changes Between groups: no significant difference	↓
Shete et al (71) ^a^	Yoga (3 months, 6 days/week)	3 months/1	IG: significant reduction CG: no significant change Between groups: no significant difference	↓↓
Geus et al (75) ^b^	Cycling to work (1 year, at least 3 times/week)	12 months/2	IG: no significant changes CG: no significant changes Between groups: no significant differences	↓
Skogstad et al (79) ^c^	Leisure time physical activity (8 weeks)	15 months/2	Significant reduction (at 15 months)	↓↓
Meyer et al (77) ^c^	Stair use (12 weeks)	6 months/2	No significant changes following intervention	—
**Fibrinogen**				
Korshøj et al (68) ^a^	Aerobic exercise (indoor biking/running, 12 months, 2 times/week)	12 months/1	IG: no significant change CG: significant increase Between groups: no significant difference	—
**IL-6**				
Hewitt et al (67) ^a^	Aerobic exercise (brisk walking/light jogging, 12 weeks, 4 times/week)	12 weeks/3	IG: No significant changes CG: significant increase (week 1-4) Between groups: no significant differences	—
Shete et al (71) ^a^	Yoga (3 months, 6 days/week)	3 months/1	IG: significant reduction CG: no significant change Between groups: significant difference	↓↓
Skogstad et al (79) ^c^	Leisure time physical activity (8 weeks)	15 months/2	Significant reduction (at 15 months)	↓↓
**TNF-α**				
Hewitt et al (67) ^a^	Aerobic exercise (brisk walking/light jogging, 12 weeks, 4 times/week)	12 weeks/3	IG: significant reduction (week 1-4), non- significant reductions (week 1-8, 1-12) CG: no significant changes Between groups: no significant differences	↓
Shete et al (71) ^a^	Yoga (3 months, 6 days/week)	3 months/1	IG: significant reduction CG: no significant change Between groups: significant difference	↓↓
Skogstad et al (79) ^c^	Leisure time physical activity (8 weeks)	15 months/2	No significant changes	—

a Randomized controlled trial.

b Non-randomized study of intervention, controlled.

c Non-randomized study of intervention, uncontrolled.

**Figure 2:**
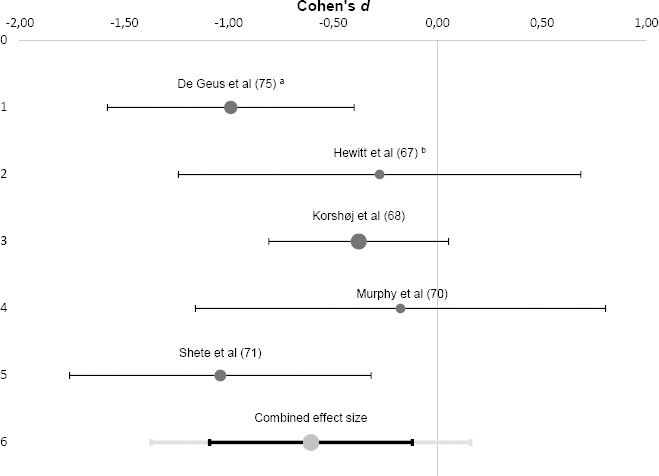
Forrest plot of individual and combined effect size(s) for workplace physical interventions and C-reactive protein. ^a^ Geus et al (75): results apply to the total study group (including men and women). ^b^ Hewitt et al (67): only the last follow up measure (after 12 weeks was considered for this meta analysis.

A meta-analysis was performed for CRP based on the five controlled studies (see figure 2). Results showed a combined effect size of Cohen’s d=-0.61 (range -1.04– -0.18) that was significantly different from zero [z(242)=-3.47, P<0.001, 95% confidence interval (CI) -1.09– -0.12]. This effect was medium-to-large in size and indicated that the physical interventions resulted in a significant reduction of workers’ CRP levels. The studies included in this pooled effect size showed no significant heterogeneity (Q=5.64, P =0.228, I^2^=29.1%). An additional fixed-effects meta-analysis revealed similar results. Exclusion of one study appraised with “serious” RoB (75) resulted in an attenuated, yet still significant negative effect (d=-0.48, 95% CI -1.04–0.08, P=0.003).

With regard to other inflammatory markers, three studies examined pro-inflammatory cytokines and two found reductions in IL-6 (71, 79) and TNF-α (67, 71), respectively.

***Mental Interventions*.** We identified five studies on mental interventions, including four RCT (65, 66, 69, 72) and one uncontrolled study (78). Two RCT scrutinized meditation- and/or mindfulness-based trainings, one among emergency department professionals (65) and one in medical residents (69). Similarly, a resilience training was examined in an uncontrolled study among law-enforcement officers (78). Hasson et al (66) evaluated a web-based health promotion tool in IT and media workers, which in the intervention group (IG) additionally included classical stress management exercises (eg, time-management, relaxation) and a chat. A cross-over study assessed recreational music-making, ie, group drumming, in male corporate employees (72). All results are presented in table S4. Concerning gene expression, findings were mixed with two significant intervention effects, ie, an upregulation of TNF-α mRNA (65) and a downregulation of IL-10 mRNA (72), and non-significant effects for other cytokine mRNA levels.

#### Organizational/Structural Interventions

We found four NRSI on organizational/structural interventions. Two studies assessed effects of work reorganization. One investigated changes in physiological markers following a major reorganization of non-state public offices (73), and another measured physiological effects of outsourcing among bus drivers (76). Wultsch et al (80) examined inflammatory effects of extended daily working times in office workers and carpenters. In addition, we included one study among university professors that investigated acute inflammatory reactions in saliva after lecturing to students (74). All results are depicted in table S5. Due to “serious” and “critical” RoB appraisals, reported findings need to be interpreted with caution. The two studies that measured employees’ CRP found significant increases after the intervention (73), yet one just in younger participants (80). For fibrinogen, no studies showed significant changes (73, 76). Regarding cytokines, for IL-6 one (73) out of two studies (80) reported significant upregulations. For other cytokines, increases were observed in response to an acute work stressor, ie, lecturing (except for IL-10) (74).

### Observational studies

Overall, seven observational studies were retrieved (table 3). Four studies (82, 83, 86, 87) applied Karasek’s job demand–control(–support) JDC(-S) model (10, 88): Job strain (82, 86, 87) and workplace social support (86, 87) were not prospectively related to CRP. Meanwhile, when the source of social support was specified, high supervisor support (in contrast to coworker support) was associated with lower CRP among women but not men (83). Job demands were not related to fibrinogen and leukocyte count (87) as well as IL-6 (82, 86). However, there were indications for small protective effects of job control regarding fibrinogen (87) and IL-6 (86) among women and leukocyte counts among men (87). For social support, Shirom et al (87) found no effects, but notably Magnusson Hanson et al (86) showed that poor workplace support – albeit weakly – was linked to higher IL-6 levels, which partially mediated the association with diabetes. Lee et al (85) investigated job stress in hospital nurses based on criteria related to the JDC(-S) model by comparing measures of objective (eg, data on staffing patterns) and subjective (ie, self-report data) stress: they identified significantly lower numbers of white blood cells in the group with high objective stress, but found no effects of subjective stress and for cytokines (85). Moreover, organizational justice and inflammation were surveyed in a large cohort study (84): Among men, but not women, low self-reported justice was associated with increased CRP and IL-6 in the long-term. Besides these model-based studies, we found one small exploratory study that compared acute effects of occupational physical demands in two groups with high (eg, construction workers) and low (ie, sedentary work) risk of work-related musculoskeletal disorders (81): IL-6 levels were greater in the high-risk group yet showed opposed temporal patterns in the two groups (table 3).

**Table 3:** Work-Related Exposures and Inflammatory Biomarkers [CRP=C-reactive protein; IFN-γ=interferon-gamma; IL=interleukin; JDC(S)=job demand-control(-support) model; NS=not significantly/ no significant; TNF-α=tumor-necrosis-factor-alpha. ↑↑ Significant positive association between working condition and inflammatory biomarker; ↑ Tendency for positive association between working condition and inflammatory biomarker, non-significant; — No significant association between working condition and inflammatory biomarker; ↓ Tendency for negative association between working condition and inflammatory biomarker, non-significant; ↓↓ Significant negative association between working condition and inflammatory biomarker; ↑↑* Significant increase in inflammatory biomarker (group comparison); ↑* Tendency for increase in inflammatory biomarker, non-significant (group comparison); —* No significant differences in inflammatory biomarkers (group comparison); ↓* Tendency for decrease in inflammatory biomarker, non-significant (group comparison); ↓↓* Significant decrease in inflammatory biomarker (group comparison).]

Marker and study	Type of exposure	Follow-up: period/number	Key findings	Direction of effect
**CRP**				
Dich et al (82)	JDC	~10-11 years/2	Job demands, decision latitude, job strain NS correlated with CRP	—
Magnusson Hanson et al (86)	JDCS	10 years/2	Job demands, job control, job strain, workplace social support NS associated with subsequent CRP	—
Shirom et al (87)	JDCS	18-22 months/1	Workload, perceived control, social support NS associated with CRP	—
Eguchi et al (83)	Source-specific workplace social support (supervisor, coworker)	1 year/1	Supervisor support significantly negatively related to CRP in women (β=-0.11, P<0.01), not significantly related to CRP in men Coworker support NS related to CRP	↓↓ (supervisor support, women)
Elovainio et al (84)	Organizational justice	~ 14 years/2	Organizational justice significantly negatively associated with CRP in men (percentage change: -4.0, P=0.02); no associations in women	↓↓ (men) — (women)
**Fibrinogen**				
Shirom et al (87)	JDCS	18-22 months/1	Workload NS associated with fibrinogen Control significantly negatively associated in females (β=-0.09, P<0.05), no associations in males Social support NS associated with fibrinogen	Workload — Control ↓↓ (women) Support —
**IFN-γ, IL-1β and TNF-α**				
Lee et al (85)	Job stress (objective and subjective job stressors: low vs. high)	8 months/8	IFN-γ: NS differences between low vs. high objective and subjective job stress	—*
			IL-1β: NS differences between low vs. high objective and subjective job stress	—*
			TNF-α: Marginally lower level of TNF-α (ng/ml) in high objective job stress group (Mdn=1.7) compared to low (Mdn=2.2, P=0.07) NS differences between low vs. high subjective job stress	↓*
**IL-6**				
Dich et al (82)	JDC	~10-11 years/2	Job strain, job demands, decision latitude NS correlated with IL-6	—
Magnusson Hanson et al (86)	JDCS	10 years/2	Social support ^a^ associated with subsequent IL-6 (β=0.03, P=0.051) Job demands and control ^a^ NS associated with subsequent IL-6 Sex stratified analyses: Job control ^a^ significantly associated to subsequent IL-6 in women (β=0.07, P<0.05), not men	Support ^a^ ↑ Demands — Control — Control ^a^ ↑↑ (women)
Christian & Nussbaum (81)	Occupational physical demands (high vs low)	1 working week/5	Higher IL-6 levels in high risk group (at all time points) Interaction time x group (F=2.53, P=0.07)	↑* ↑↓* (high) ↓↑* (low)
Elovainio et al (84)	Organizational justice	~ 14 years/2	Organizational justice significantly negatively associated with IL-6 in men (percentage change: -4.5, P=0.01); no associations in women	↓↓ (men) — (women)
**Leukocyte count**				
Lee et al (85)	Job stress (objective and subjective job stressors: low vs. high)	8 months/8	Significant lower level of white blood cells (number of cells per mm^3^) in high objective job stress group (Mdn=7.17) compared to low (Mdn=8.06, P=0.03) NS difference between low vs. high subjective job stress	↓↓*
Shirom et al (87)	JDCS	18-22 months/1	Workload NS associated with leukocyte count Control significantly negatively associated in males (β=-0.06, P<0.05), NS associated in females Social support NS associated with leukocyte count	Demands — Control ↓↓ (men) Support —

a Higher values in the scales for workplace social support and job control indicated lower social support and lower control, respectively (86).

### Risk of bias across studies

Concerning the meta-analysis, the funnel plot indicated symmetry in the distribution of individual effect estimates suggesting absence of bias and heterogeneity (89). As less than ten studies were included, tests for asymmetry were not used (90). Based on the Trim-and-Fill method, no studies were missing to the right of the mean, so the combined effect size did not have to be adjusted for publication bias (see figure S1). Our self-rating of the overall confidence in the results per AMSTAR-2 (62) was “high”, indicating that the review provides an accurate and comprehensive summary of available studies addressing our research question (AMSTAR-2 evaluation sheet available upon request).

## Discussion

Sustained systemic low-grade inflammation has been identified as one of the major pathophysiological pathways linking exposure to chronic stress and development of severe long-term diseases. A thorough and evidence-based understanding of the role of work stress exposure for inflammatory pathways is thus imperative to develop effective prevention and mitigation measures in occupational stress research. To the best of our knowledge, this is the first systematic review and meta-analysis on associations of working conditions and chronic low-grade inflammation merely based on prospective studies. By building on a higher quality of evidence, this review advances our knowledge on effects of work stressors on chronic low-grade inflammation in employees.

Overall, 23 studies met our inclusion criteria. The extant study base was fragmented with high heterogeneity in assessed exposures and interventions. We identified four clusters of study types, ie, individual-directed/behavioral (including physical and mental) and organization-directed/structural interventions as well as observational studies.

For workplace *physical interventions* (k=7), the majority of studies reported reductions in inflammation-related plasma molecules. These interventions primarily aimed at changing individual behavior by adding physical exercises or activity into employees’ work routine (both on- and off-the-job) and were conducted among both sedentary and manual workers. The qualitative finding was corroborated in our meta-analysis demonstrating a medium to strong negative effect of physical exercise interventions (aerobic exercise, walking, yoga, cycling to work) on employees’ CRP levels (d= -0.61; k=5). This resonates well with a previous meta-analysis on studies in non-occupational settings showing that exercise training was associated with a decrease in CRP (91). Our results suggest that exercise interventions are an effective measure to reduce low-grade inflammation in employees.

Concerning *mental interventions* in the workplace (eg, stress reduction programs, music making), the study base (k=5) was limited and inconclusive. However, there were indications for changes of inflammation-related processes on intra-cellular level, ie, gene expression (65, 72) and transcription factors (69). These interventions were also individual-oriented, ie, they aimed at influencing mental processes by providing employees opportunities and skills for increasing their well-being, alleviating stress, facilitating relaxation, strengthening resilience etc. Reviews outside work settings have shown associations of psychosocial interventions, especially cognitive behavior therapy and combined psychotherapeutic interventions, with enhanced immune system function (92). In addition, salutogenic effects of mindfulness meditation and yoga practices in combination with mindfulness-based stress reduction regarding specific inflammatory markers have been suggested (93, 94). Yet, these studies included heterogeneous populations, also clinical samples, which can lead to spurious estimates of effects. Our synthesis suggests that in occupational settings, individual/behavioral interventions appear to be viable measures to ameliorate dysregulated inflammatory processes, however extended research into workplace mental interventions is warranted.

The study base on *organizational/structural interventions* was confined, with high RoB (k=4). Despite indications of responsiveness of CRP and cytokines to organizational changes (73, 80), definite conclusions would be premature. Given the high variety of organizational-level workplace interventions and differentiated effects on employee health, further investigations into particular types of organizational interventions, such as work reorganization or work time-related conditions, and their effects on inflammatory markers are necessary (95).

The majority of *observational studies* (k=7) was based on the JDC(-S) model. Results showed predominately null and/or weak associations. However, there were some indications for beneficial functions of job control and workplace social support as well as for sex-related effects. Conclusions of previous reviews are somewhat conflicting: Whereas Nakata (37) suggested that inflammatory markers might be less sensitive to job strain, Wright et al (38) inferred that workplace stress is positively associated with CRP, especially when measured with the JDC model. Despite the evidence for a close link between personal relationships, including social support amongst others, and immune function (96), we found only three studies on workplace social support and inflammatory outcomes. Consistent with previous reviews we deem future research into resources and potentially beneficial effects of workplace support of particular interest (37).

We also sought to detect studies examining stress reactions in terms of inflammation evoked by work-related ICT use. Ultimately, we identified just one study showing that the application of a web-based health promotion tool modulated TNF-α (66). The extent to which working conditions associated with ICT use or respective workplace interventions affect inflammatory processes needs thus to be further investigated.

### Work stress and inflammation: methodological and conceptual considerations

For the interpretation of the collected evidence, some pivotal aspects potentially influencing associations of working conditions and inflammation warrant attention. First, included studies differed tremendously in time lags of follow-ups, spanning a few hours to 14 years, and in numbers, ranging from one to eight follow-up assessments. In longitudinal research the magnitude of effects might vary with the span of the follow-up, ie, whether it corresponds with the true underlying time lag of the outcome under study (43). Multi-wave designs increase the likelihood of detecting effects compared to two-wave designs, and response latencies of respective outcomes may depend on type, intensity, and duration of exposures as well as context factors (43, 97). Thus, differences in follow-up measurements of inflammatory markers may help to explain the disparity in findings of the present studies.

Moreover, although longitudinal designs are suggested to overcome the problems of cross-sectional designs in examining causality, reversed or reciprocal causation and third variables constitute critical issues in longitudinal research (98). As for the question of reverse effects, we are aware of only two of the included observational studies that also tested for associations in the opposite direction in full panel designs, ie, inflammatory markers on subsequent appraisal of working conditions (86, 87). Concerning influences of third variables, many studies controlled for variables critical in stress physiology, such as sex, age, health behaviors (eg, physical activity, smoking), body mass index, (hormone) medication, and baseline levels of respective markers. However, studies differed in the selection and number of included covariates, entailing varying degrees of threats to internal validity (see also tables S2 and S3). For the investigation of cause–effect relationships between intervention and outcome, RCT are considered the gold standard; yet this design is often not feasible in occupational settings (99). Notwithstanding, half of the identified interventions were RCT, so we were able to draw our meta-analysis upon a high level of evidence, yet confined study base.

Furthermore, inflammation should not be assessed in isolation with regard to stress but in the light of potential disruptions of interactions and feedback loops with other stress axes. Inflammation is affected by the two major stress systems HPA axis and ANS through complex neuroendocrine-immune cascades and interactions, indicating that the effects of stress system mediators on the inflammatory system are not linear (100). There is consistent evidence that chronic stress is related to alterations in the sensitivity of target tissue to stress signals, most importantly glucocorticoid resistance, which is associated with increases in circulating inflammatory mechanisms (100). Examples of these complex multi-system interdependences are the – at first glance surprising – results of Dunne et al (65), where TNF-α mRNA increased in the IG, and Hasson et al (66), where TNF-α decreased in the CG. Both authors provided post-hoc explanations concerning potentially impaired negative feedback loops with the HPA axis in chronic stress, and Dunne et al proposed that the observed increase could be due to decreases in cortisol following stress reduction in the IG. Anti-inflammatory effects of cortisol have been well-described (100).

Lastly, we focused on working conditions, as they are more modifiable to workplace interventions than personal factors. Nonetheless, we acknowledge that employees’ intrinsic characteristics, such as personal resources, affective-cognitive states, and coping styles, play a significant role in (work) stress perception and regulation (101–103). For instance, higher work engagement was found to be associated with lower subsequent high-sensitivity CRP (104), whereas over-commitment was associated with reduced immunity (39).

### Limitations and strengths

Our findings should be interpreted in the light of some limitations. By defining the PECOS/PICOS components, we might have excluded relevant aspects. For example, shiftwork is an important risk factor for inflammation, and in our search, we found sound interventional studies in shift worker samples (eg, 105). However, as it is difficult to disentangle effects of working conditions from the effects of circadian misalignment per se on inflammatory markers (106), we decided a priori to exclude these studies. Moreover, for greater external validity, we only considered investigations in real-world occupational settings. Notwithstanding, we are aware of high-quality laboratory studies on stress responsiveness in chronic work stress (107, 108) and simulation studies in high-risk professions, such as firefighting (109). The generalizability of our findings is restricted to the working population, yet we included a broad range of different professional and occupational groups. A main limitation of our review is the limited study base with great heterogeneity regarding intervention contents and modes of implementation, work exposures, and occupational sectors. By clustering studies following an inductive logic we attempted to build more homogenous subgroups of studies. However, the disparity of clusters in combination with the scarcity of data currently limits the possibility and adequacy of deriving overall conclusions. We acknowledge, that some employer-instigated health promotion approaches were not limited to the workplace and included components to be performed off site/ off duty or on the way to work (eg, cycling to work). This may have introduced heterogeneity within our clusters and impeded a clear differentiation concerning the nature and implementation of included interventions as well as ensuing inferences concerning effectiveness. An important strength of our investigation is that we developed and determined our methodology prior to the start in a peer-reviewed protocol, limiting the risk of reporting bias and ensuring higher quality. Further strengths pertain to our pre-defined classification system, which enabled us to draw conclusions per cluster given the high heterogeneity of identified studies, and the consideration of a comprehensive set of inflammatory biomarkers. Furthermore, we applied rigorous and thorough RoB assessments in and across studies, allowing for a critical evaluation of the presented evidence.

### Implications for occupational health management and future research

For occupational health management, a holistic approach integrating both individual/behavioral and organizational/structural measures may generate greater benefits for employee health (110). The reported physical interventions primarily aimed at modifying employees’ behaviors, ie, increasing their physical activity to counteract predominantly sedentary work or high aerobic workload (eg, cleaning). Yet, rather than merely reducing symptoms of work-related strain, preventive measures on an organizational level that target the sources of strain are crucial (111). Primary preventive interventions address stressors through changes in the psychosocial working conditions, physical work environment, or organization and include for instance enhancement of social support or autonomy, and job redesign (110, 112, 113). Although we did not find intervention studies directly aiming at modifying psychosocial work stressors, pooled results of the observational studies suggesting protective effects of job control and social support to employees’ inflammation indicate that these factors could be important leverage points to future intervention studies. Furthermore, all retrieved interventions referred to unidimensional approaches, what points to the need for evaluation of complementary, multi-component interventions consisting of individual and organizational measures regarding effects on physiological stress parameters (eg, 114.).

In the light of our findings and further considerations, we suggest the following avenues for future research: First and foremost, more prospective studies are needed. For workplace interventions, RCT or at least controlled studies on mental, physical, and organizational interventions are necessary. In observational research, deployment of full cross-lagged panel designs provides reliable insights into the direction of effects. Second, future research should investigate combinations of work exposures, eg, both psychosocial work factors and occupational physical activity. Investigations into potential additive and interactive effects on psychophysical health might better reflect real-world occupational situations. Moreover, we advocate a clear conceptual and methodological differentiation between objective work exposures on the one hand and subjective reactions of the individual workers on the other. For research on psychosocial work stress however, this is often not feasible, as per definition, psychosocial factors at work concern *interactions* between both work environment, job content, organizational conditions and individual factors of the workers, such as capacities, needs etc., which may influence health and well-being (115). Third, future research should examine effects of work-related ICT use on inflammation in occupational settings with high-quality designs. With the dynamic advancement of digitalization and technologization of humans’ workplaces, research into the concept of technostress (116–118) has been rapidly increasing. However, physiological effects associated with technostress are under-researched (40), and assessment of inflammatory markers might reveal valuable insights into potential detrimental health effects. Forth, while CRP and cytokines were surveyed most frequently, future research should consider further biomarkers of inflammatory processes (such as cellular and intracellular) and interactions with other stress systems. This will contribute to a deeper understanding of pathophysiological pathways from work stress exposure to disease.

### Concluding remarks

This systematic review and meta-analysis on associations of working conditions and chronic low-grade inflammation showed that the current base of prospective studies is limited and diverse in methodology, exposures, and inflammatory outcomes. Meta-analytic evidence was established for workplace physical exercise interventions, which were found to significantly reduce employees’ CRP level. Complementary to previous reviews mainly based on cross-sectional studies, our review revealed a more differentiated picture of potential associations, suggesting that at this stage, definite conclusions are premature. The review identified important research gaps and derived recommendations for future high-quality studies to advance knowledge in this field. Concerning occupational health management practice, we conclude that physical activity interventions for employees are effective counter-measures to chronic low-grade inflammation.

### Funding

The review is part of the research project “*Identifikation biomedizinischer und gesundheitlicher Wirkweisen von positiven und negativen Auswirkungen von digitalem Stress und dessen Bewältigung*” [‘*Identification of biomedical and health-related modes of action of positive and negative effects of digital stress and coping with it’*] which is part of the Bavarian Research Association on Healthy Use of Digital Technologies and Media (ForDigitHealth), funded by the Bavarian Ministry of Science and Arts. MW and DN have been partly funded by the Munich Centre for Health Sciences (MC-Health). The funders were not involved in the study design, the collection, analysis and interpretation of the data, the writing of the report, and the decision to submit the paper for publication.

The authors declare no conflicts of interest.

## Supplementary material

Supplementary material
