# Supplementary material for: Associations of working conditions and chronic low-grade inflammation among employees: a systematic review and meta-analysis
Source: Scand J Work Environ Health. 2021 Oct 31;47(8):565–81. doi: 10.5271/sjweh.3982 (PMC9058622; doi:10.5271/sjweh.3982)

Associations of working conditions and chronic low-grade inflammation among employees: a systematic review and meta-analysis[1]

by Helena C Kaltenegger, MSc,[2] Linda Becker, PhD, Nicolas Rohleder, PhD, Dennis Nowak, MD, Matthias Weigl, PhD

1. *Supplementary Material*
2. *Correspondence to: Helena C. Kaltenegger, MSc, Institute and Clinic for Occupational, Social and Environmental Medicine, University Hospital, LMU Munich, Ziemssenstraße 1, 80336, München, Germany. [E-mail: helena.kaltenegger@med.uni-muenchen.de]*

Table S1 Outcome Category, Definition, and Included Inflammatory Biomarkers per Category
Table S2 Extracted Data and Risk of Bias Assessment per Study
Table S3 Results of the Risk of Bias Assessment (for Randomized Controlled Trials and Non-Randomized Studies of Interventions) and Quality of Reporting Assessment (for Observational Studies)
Table S4 Workplace Mental Interventions and Inflammatory Biomarkers
Table S5 Organizational/ Structural Interventions and Inflammatory Biomarkers
Figure S1 Funnel Plot for Studies on Workplace Physical Interventions and C-reactive Protein

Table S1

Outcome Category, Definition, and Included Inflammatory Biomarkers per Category.

**Outcome category**	**Definition of outcome category**	**Inflammatory biomarkers (per outcome category)**
**Cells**	Inflammation-related processes on cell level as a component of cellular immunity	Leukocytes<br>Eosinophils<br>Granulocytes<br>Lymphocytes<br>Macrophages<br>Monocytes<br>Neutrophils<br>Dendritic cells
**Plasma molecules**	Inflammation-related processes on plasma protein level as a component of humoral immunity	Acute-phase proteins<br>C-reactive protein (CRP)<br>Fibrinogen<br>Serum amyloid A<br>Cytokines<br>Chemokines<br>Interferon-gamma (IFN-γ)<br>Interleukins (IL)<br>Lymphokines<br>Monokines<br>Tumor necrosis factor-alpha (TNF-α)<br>Cell-free DNA<br>Inflammasomes<br>Intercellular adhesion molecule-1
**Intracellular processes**	Inflammation-related processes on intracellular level	Transcription factors<br>AP-1<br>NF-IL6<br>NF-kappa B<br>Gene expression<br>Transcripts for proteins associated with inflammatory processes<br>Transcriptomics focusing on or revealing inflammatory processes

Table S2

Extracted Data and Risk of Bias Assessment per Study

available at: https://osf.io/u2s9p/

Table S3

Results of the Risk of Bias Assessment (for Randomized Controlled Trials and Non-Randomized Studies of Interventions) and Quality of Reporting Assessment (for Observational Studies)

**Study (type)**	**Risk of bias domains**							**Overall risk of bias**
**RCT**	Randomization process			Deviations from intended interventions	Missing outcome data	Measurement of the outcome	Selection of the reported result	
Dunne et al (65)	+			+	±	+	±	±
Hasson et al (66)	+			+	+	+	±	±
Hewitt et al (67)	+			+	+	+	±	±
Korshøj et al (68)	±			+	±	+	+	±
Lebares et al (69)	+			+	+	+	±	±
Murphy et al (70)	+			+	±	+	±	±
Shete et al (71)	+			+	±	+	±	±
Wachi et al (72)	±			+	+	+	±	±
**NRSI**	Confounding	Selection of participants into the study	Classification of interventions	Deviations from intended interventions	Missing data	Measurement of outcomes	Selection of the reported result	**Overall risk of bias**
Controlled:								
Carlsson et al (73)	±	+	+	+	–	+	+	–
Filaire et al (74)	–	±	+	+	n.i.	+	+	–
Geus et al (75)	–	+	+	±	n.i.	+	+	–

Table S3 continued

**Study (type)**	**Risk of bias domains**							**Overall risk of bias**
**NRSI**	Confounding	Selection of participants into the study	Classification of interventions	Deviations from intended interventions	Missing data	Measurement of outcomes	Selection of the reported result	
Netterstrøm & Hansen (76)	−	+	+	+	− −	+	+	− −
Uncontrolled:								
Meyer et al (77)	− −	+	n.a.	+	+	+	+	− −
Ramey et al (78)	− −	±	+	+	±	+	+	− −
Skogstad et al (79)	−	±	+	+	±	+	+	−
Wultsch et al (80)	− −	+	+	+	n.i.	+	+	− −

**Observational**	**Quality of reporting** Number of criteria reported	Number of relevant criteria	**Summary score**
Christian & Nussbaum (81)	13	32	0.41
Dich et al (82)	23	33	0.70
Eguchi et al (83)	22	33	0.67
Elovainio et al (84)	23	33	0.70
Lee et al (85)	18	33	0.55
Magnusson Hanson et al (86)	21	33	0.64
Shirom et al (87)	22	33	0.67

Note. RCT = randomized controlled trial; NRSI = non-randomized study of intervention.

RCT: '+' = low risk of bias; '±' = some concerns; '−' = high risk of bias

NRSI: '+' = low risk of bias; '±' = moderate risk of bias; '−' = serious risk of bias; '− −' = critical risk of bias; 'n.i.' = no information; 'n.a.' = not applicable

Observational: summary score = number of criteria reported divided by number of relevant criteria; scale range: 0-1 (higher scores indicate better quality)

Table S4

Workplace Mental Interventions and Inflammatory Biomarkers

Marker	Study	Type of mental intervention (duration, frequency)	Follow-up: period/number	Key findings	Direction of effect
CRP	Ramey et al (78)[b]	Resilience training (~ 14-15 weeks; one educational class, one telementor session, 3 months practice in the field)	6 months/1	No significant change following intervention	—
TNF-α	Hasson et al (66)[a]	Web-based stress management and health promotion tool (6 months, daily/ regular usage)	6 months/1	Significant decrease in reference group compared to IG	↓↓*
Gene expression:					
TNF-α mRNA	Dunne et al (65)[a]	Attention-based training program (7 weeks, 4 sessions à 4 hours)	9 weeks/1	IG: significant increase<br>CG: no significant change	↑↑
IL-6 mRNA				No significant changes (both groups)	—
	Wachi et al (72)[a]	Recreational music making (1 hour)	3 hours/1	IG: no significant change<br>CG: no significant change<br>Between groups: no significant differences	—
IL-2 mRNA				IG: no significant change<br>CG: no significant change<br>Between groups: no significant differences	—
IL-10 mRNA				IG: significant decrease<br>CG: no significant change<br>Between groups: significant differences	↓↓
IFN-γ mRNA				IG: no significant change (phases 1 & 2)<br>CG: no significant change (phase 1); significant increase (phase 2)<br>Between groups: no significant differences (phase 1); significant difference (phase 2)	↓

Table S4 continued

**Marker**	**Study**	**Type of mental intervention (duration, frequency)**	**Follow-up: period/number**	**Key findings**	**Direction of effect**
Transcription factors:					
AP-1	Lebares et al (69)[a]	Enhanced Stress Resilience Training (ESRT; tailored mindfulness-based stress reduction; aggregated data of two trials; ESRT-1: 8 weeks, 120 minutes/ week; ESRT-2: 6 weeks, 90 minutes/ week)	6-8 weeks/1	IG: significant reduction<br>CG: significant increase	↓↓
NF-kappaB				IG: significant reduction<br>CG: no significant change	↓↓
Leukocyte count	Wachi et al (72)[a]	Recreational music making (1 hour)	3 hours/1	IG: significant increase (phase 1); no significant change (phase 2)<br>CG: no significant change (phase 1); significant increase (phase 2)<br>Between groups: no significant differences (phase 1); significant difference (phase 2)	↑↑ (phase 1)<br>↑↑* (phase 2)

Note. CG = control group; CRP = C-reactive protein; IL = interleukin; IG = intervention group; TNF-α = tumor-necrosis-factor-alpha.
[a] Randomized controlled trial
[b] Non-randomized study of intervention, uncontrolled

↑↑ Significant increase in inflammatory biomarker following intervention (and no significant change/ decrease in control)

↑ Tendency for increase in inflammatory biomarker, non-significant

— No significant difference(s) in inflammatory biomarker (between groups/ within group)

↓ Tendency for decrease in inflammatory biomarker, non-significant

↓↓ Significant decrease in inflammatory biomarker following intervention (and no significant change/ increase in control)

↑↑* Significant increase in inflammatory biomarker in control group (and no significant change in intervention group)

↓↓* Significant decrease in inflammatory biomarker in control group (and no significant change in intervention group)

Table S5

Organizational/ Structural Interventions and Inflammatory Biomarkers

Marker	Study	Type of mental intervention and characteristics	Follow-up: period/number	Key findings	Direction of effect
CRP	Carlsson et al (73)[a]	Workplace reorganization (merger, new job)	2 years/1	Merger: significant increase<br>New job: no significant change (compared with CG, respectively)	↑↑ (merger)<br>↑ (new job)
	Wultsch et al (80)[b]	Extended working periods (from 8 to 12 hours/day, 3 months)	3 months/1	No significant change (total sample)<br>Significant increase in younger participants (21-30 years)	— (total sample)<br>↑↑ (young participants
Fibrinogen	Carlsson et al (73)[a]	Workplace reorganization (merger, new job, control)	2 years/1	Merger: no significant change<br>New job: no significant change (compared with CG, respectively)	—
	Netterstrøm & Hansen (76)[a*]	Outsourcing	13 months/2	No significant changes after intervention	—
IL-6	Carlsson et al (73)[a]	Workplace reorganization (merger, new job, control)	2 years/1	Merger: significant increase<br>New job: significant increase (compared with CG, respectively)	↑↑ (merger)<br>↑↑ (new job)
	Wultsch et al (80)[b]	Extended working periods (from 8 to 12 hours/day, 3 months)	3 months/1	No significant change	—
IL-2	Filaire et al (74)[a**]	Lecture to 200 students (compared to a working day without lecture)	2 working days/3	Significant increase after lecture	↑↑
IL-4				Significant increase after lecture	↑↑
IL-10				No significant effects	—
TNF-α				Significant increase after lecture	↑↑

Note. CG = control group; CRP = C-reactive protein; IL = interleukin; TNF-α = tumor-necrosis-factor-alpha.

[a] Non-randomized study of intervention, controlled

[b] Non-randomized study of intervention, uncontrolled
* Controlled for baseline only
** Within-subject design

↑↑ Significant increase in inflammatory biomarker following intervention

↑ Tendency for increase in inflammatory biomarker, non-significant

— No significant change in inflammatory biomarker

↓ Tendency for decrease in inflammatory biomarker, non-significant

↓↓ Significant decrease in inflammatory biomarker following intervention

Figure S1

Funnel Plot for Studies on Workplace Physical Interventions and C-reactive Protein

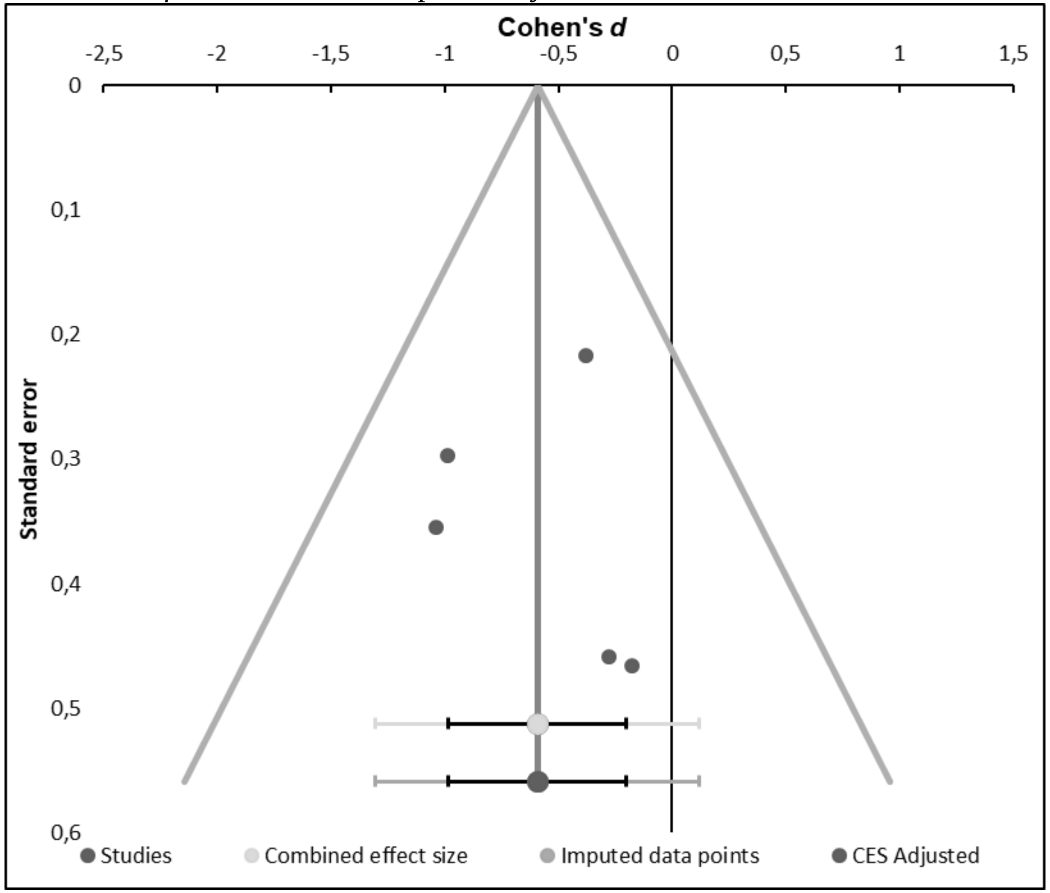

Supplement: Supplementary material [file SJWEH-47-565-S001.pdf]
